# Association d’une cytostéatonécrose néonatale, d’une hypertriglycéridemie et d’une hypercalcémie: à propos d’une observation

**Published:** 2012-02-16

**Authors:** Rachid Abilkassem, Nezha Dini, Mohamed Oukabli, Mohamed Kmari, Aomar Agadr

**Affiliations:** 1 Service de pédiatrie, Hôpital Militaire d’Instruction Mohamed V, Rabat, Maroc; 2 Service d’anatomie pathologique, Hôpital Militaire d’Instruction Mohamed V, Rabat, Maroc

**Keywords:** Cytostéatonécrose néonatale, hypercalcémie, hypertriglycéridemie, Maroc

## Abstract

La cytostéatonécrose du nouveau-né est une hypodermite aigue qui apparaît dans les premières semaines de vie. Nous rapportons les caractéristiques cliniques et histologiques d’une cytostéatonécrose chez un nourrisson âgé de trois semaines, admis pour des lésions cutanées à type de placards sous-cutanées dures, localisées sur le dos. Le nouveau-né a développé une hypercalcémie et une hypertriglycéridemie d’évolution favorable sous traitement symptomatique de même que les lésions cutanées qui ont disparu en quelques semaines.

## Introduction

La cytostéatonécrose du nouveau-né (CSNN) est une hypodermite aigue qui apparaît entre la première et la sixième semaine de vie. C’est une affection généralement bénigne. Parfois elle se complique d’une hypercalcémie qui peut être grave si elle n’est pas prise en charge à temps [1]. Un terrain de dyslipidémie familiale constitue un facteur de risque de son apparition.

### Patient et observation

**Figure 1:**
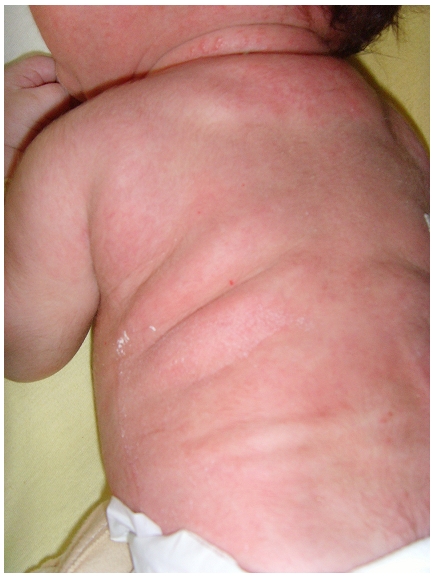
Placards et nodules sous-cutanés localisés au niveau du dos

**Figure 2:**
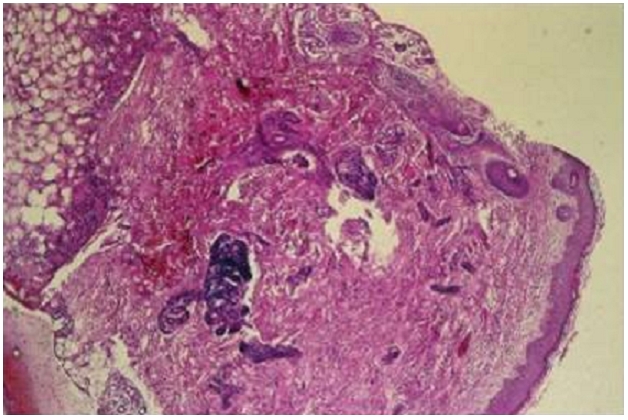
Panniculite lobulaire hypodermique (HEx40)

**Figure 3:**
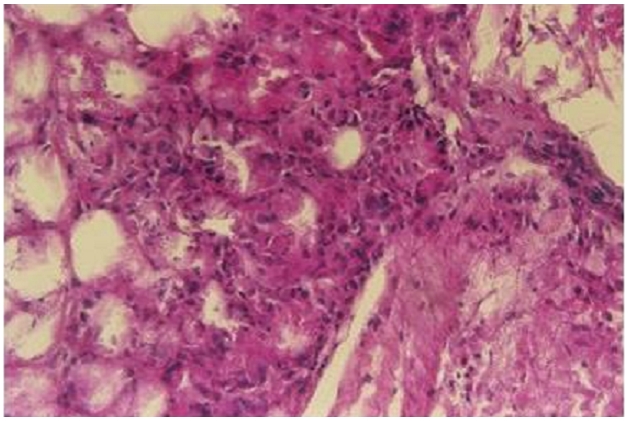
Infiltrat inflammatoire composé d’histiocytes, de cellules géantes et quelques lymphocytes. Noter les images en aiguilles cristallines à la périphérie de certains adipocytes suggestifs des cristaux dissous (HEx400)

Nouveau-né de sexe masculin admis à trois semaines de vie pour prise en charge de nodules cutanés siégeant au niveau du dos associés à des vomissements. L’examen clinique retrouve un nouveau-né apyrétique, tonique, réactif, sans signe de détresse vitale, l’examen des téguments notent des lésions cutanées à type de placards sous-cutanés durs, légèrement douloureux, de couleur rouge-violine, localisées au niveau du dos évocatrices d’une cytostéatonécrose (Figure 1). Une biopsie de peau confirme le diagnostic (Figure 2, Figure 3) en montrant un infiltrat inflammatoire composé d’histiocytes, de cellules géantes et quelques lymphocytes et des images en aiguilles cristallines à la périphérie de certains adipocytes suggestifs des cristaux dissous. Dans les antécédents, on note une naissance à terme par voie basse avec notion de souffrance néonatale rapidement résolutive. Le bilan à l’admission retrouve une hypercalcémie à 110 mg/l. La phosphorémie et l’albuminémie sont normales, la 1,25 – dihydroxy-vitamine D est élevé à 258 pmol/l (43-148 pmol/l) et la parathormone effondrée à 3 pg/ml (14-72). Le bilan lipidique montre une hypertriglycéridemie chez le nouveau-né et sa mère respectivement à 3,16 g/l et 4,46 g/l pour une valeur normale (0,6-1,5 g/l). L’hémogramme n’a pas objectivé d’anémie ou de thrombocytose. L’échographie rénale n′a pas trouvé de néphrocalcinose.

Le traitement a consisté en un régime pauvre en calcium, une hyperhydratation saline parentérale associée à un diurétique de l’anse (furosémide/Lasilix™) pendant 8 jours consécutifs permettant une normalisation durable de la calcémie et une régression complète des signes cutanés dès l’âge de deux mois. L’allaitement maternel et la supplémentation vitamino-calcique ont pu être réintroduis.

## Discussion

La cytostéatonécrose du nouveau-né est une panniculite rare, dont la fréquence est difficile à estimer. Elle survient chez le nouveau-né à terme ou post terme, macrosome, aux antécédents d′hypoxie, d′hypothermie, d′extraction instrumentale ou de césarienne [1-3]. Des facteurs maternels tels que le diabète gestationnel, la pré-éclampsie, la prise de cocaïne ou d’inhibiteurs calciques sont rapportés. Un terrain lié à l’enfant a été suspecté dans plusieurs observations tel une anémie [4] ou une thrombocytose [5]. Ces deux facteurs pourraient être responsables d’une hypoxie périphérique. Un autre facteur a été discuté dans d’autres publications et confirmé par notre cas est l’existence d’une dyslipidémie familiale. Ces observations décrivent des hypertriglycéridemie majeures lors de CSNN chez des enfants issus de mères atteintes de dyslipidémie [2,6–8], faisant discuter des anomalies du métabolisme des graisses dans la physiopathologie de la CSNN. Noojin et al [9] en 1949 a constaté que les cristaux au niveau des adipocytes et des cellules géantes multinucléées étaient composé de triglycérides, ces résultats ont été confirmés par Horsfield et Yardley en 1965 [10]. Le taux élevé des triglycérides peut-être dus à la mobilisation des acides gras des tissus graisseux. Chez notre patient l’hypertriglycéridémie était passagère. Le taux des triglycérides est revenu spontanément à la normale après disparition des lésions cutanées.

La CSNN se manifeste par des nodules sous cutanés douloureux avec placards érythémateux apparaissent en moyenne quatre jours après la naissance sur la face, les fesses, le tronc ou la racine des membres. Il s′agit de panniculite lobaire avec nécrose des adipocytes, infiltrat des cellules granulomateuses et dépôt de caséum. Le diagnostic différentiel se pose avec le sclérème néonatale, l′érysipèle.

La complication majeure est l′hypercalcémie qui survient dans un tiers des cas environ pourrait s′expliquer par la majoration de la résorption osseuse, ou par synthèse de 1,25 dihydroxycholecalciférol par les cellules granulomateuses. Dans notre cas il semblerait que le deuxième mécanisme soit en cause bien que les marqueurs de résorption osseuse n′aient pas été recherchés. L′hypercalcémie pouvant conduire à des complications cardiaques, neurologiques ou rénales (néphrocalcinoses et les lithiases) parfois létales [11,12]

Le traitement de la CSNN n′est pas codifié, il est essentiellement symptomatique (réchauffement, réhydratation). Le traitement conventionnel en cas d′hypercalcémie sévère associe une hyperhydratation avec du sérum salé isotonique (3 ml/m^2^/j), des diurétiques hypercalciuriants tels que les diurétiques de l’anse, des corticoïdes à faible dose, et un régime pauvre en calcium et en vitamine D. Ce traitement ne permet pas toujours de normaliser la calcémie. L’utilisation des biphosphonates a montré son efficacité dans quelques cas [2,3,12].

Chez notre malade nous avons opté pour un traitement par hyperhydratation saline et diurétique avec une évolution favorable et les surveillances des calcémies hebdomadaires sont normales.

## Conclusion

La cytostéatonécrose est une entité de diagnostic clinique. La dyslipidémie maternelle constitue un nouveau facteur de risque pour son apparition. La complication principale est l’hypercalcémie qui doit être recherchée systématiquement pendant les premières semaines de vie afin d’instaurer un traitement optimal et éviter une évolution qui peut être fatale.
